# Novel discoveries targeting gemcitabine‐based chemoresistance and new therapies in pancreatic cancer: How far are we from the destination?

**DOI:** 10.1002/cam4.2384

**Published:** 2019-09-01

**Authors:** Wenhao Luo, Gang Yang, Jiangdong Qiu, Jingyang Luan, Ying Zhang, Lei You, Mengyu Feng, Fangyu Zhao, Yueze Liu, Zhe Cao, Lianfang Zheng, Taiping Zhang, Yupei Zhao

**Affiliations:** ^1^ Department of General Surgery Peking Union Medical College Hospital, Chinese Academy of Medical Sciences and Peking Union Medical College Beijing China; ^2^ Department of Vascular Surgery Zhongshan Hospital, Institute of Vascular Surgery, Fudan University Shanghai China; ^3^ Department of Oncology The Second Xiangya Hospital, Center South University Changsha China; ^4^ Department of Nuclear Medicine Peking Union Medical College Hospital, Chinese Academy of Medical Sciences and Peking Union Medical College Beijing China; ^5^ Clinical Immunology Center Chinese Academy of Medical Sciences and Peking Union Medical College Beijing China

**Keywords:** chemistry, drug therapy, metabolism, pancreatic cancer, prevention

## Abstract

Pancreatic cancer (PC) remains one of the deadliest malignancies worldwide. Chemoresistance is a significant clinical problem in pancreatic ductal adenocarcinoma (PDAC) and numerous potential mechanisms have been demonstrated but much remains to be understood. To overcome the existing limitations in PC treatment, newer approaches targeting intrinsic or acquired mechanisms have been found to improve drug therapeutic effectiveness in PC patients. Here, we provide an update of the most recent findings and their implications for clinicians, and attempt to summarize the various aspects of different individualized novel therapies for PC that could most benefit metastatic PDAC patients.

## Graphical abstract

The latest treatment schemes based on various mechanisms of drug resistance in pancreatic cancer; 2. The advantages and disadvantages of those novel therapies and enumerated feasible methods to improve the efficacy of chemo‐therapies which are hopefully applied to clinics.

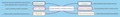

## 1 INTRODUCTION

Pancreatic cancer (PC) is the fourth leading cause of cancer deaths worldwide.^1^ The most effective treatment is surgical resection with neoadjuvant therapy. In the early stages of the disease, the few clear clinical signs or symptoms make it difficult to diagnose. Therefore, only 10%–15% of patients can undergo surgical resection of tumor tissue.^2^ Nowadays, most patients receive chemotherapy. Several major chemotherapy regimens include gemcitabine (GEM) mono‐therapy,^3^ the FOLFIRINOX scheme (oxaliplatin, irinotecan, fluorouracil, and leucovorin),^4^ GTX (GEM, docetaxel, capecitabine),^5^ GEM along with cisplatin, and the nanoparticle abraxane or albumin‐bound (nab) paclitaxel along with GEM.^6^ Currently GEM‐based chemotherapy remains the standard treatment. GEM is a deoxycytidine analog that inhibits DNA replication and thereby arrests tumor growth. However, resistance caused by various factors greatly limits the use of GEM. To overcome chemoresistance in pancreatic ductal adenocarcinoma (PDAC), several novel therapeutic approaches, including noncoding RNA, nanoparticles and liposome drugs, chemoresistance‐related signaling pathway antagonists, immunotherapy, precise therapy based on molecular types, and specific antibiotics to bacterial drug‐activated enzyme are currently being developed.

In this article, we mainly focus on the latest therapeutic targets and drugs to overcome chemoresistance in PDAC therapy.

## 2 NOVEL TREATMENTS FOR METABOLISM OF GEM

GEM (2',2'‐difluoro 2'‐deoxycytidine, dFdC) is phosphorylated to its main active triphosphate metabolite 2′,2′‐difluorodeoxycytidine triphosphate (dFdCTP) by deoxycytidine kinase (dCK), which competes with deoxycytidine triphosphate (dCTP) as an inhibitor of DNA polymerase and inhibits DNA synthesis. It is inactivated mainly by cytidine deaminase (CDA) to 2,2′‐difluorodeoxyuridine (dFdU).^7^

It is reported that paclitaxel (PTX) can inhibit the expression of CDA to enhance the antitumor efficiency of GEM. Meng et al reported a lipid bilayer (LB)‐mesoporous silica nanoparticle (MSNP) that can codeliver a synergistic GEM/PTX combination and decrease the expression of CDA, showing the most effective function of shrinking cancer mass in an orthotopic pancreatic cancer model than free GEM and GEM plus abraxane.^8^

The two subunits of ribonucleotide reductase (RR), RRM1, and RRM2, are responsible for the conversion of ribonucleosides to deoxyribonucleoside triphosphates (dNTPs), which are essential for DNA repair. They are known as GEM resistance genes and affect its metabolism. A study reported that gambogic acid, one of the main components of gamboge, sensitizes GEM efficacy in pancreatic cancer by reducing the expression of RRM2.^9^ Another study proved that transfection of *miR‐608* or *miR‐101‐3p* leads to decreased expression of RRM1 in GEM‐resistant PDAC cells.It suggests that their codelivery with GEM has a more effective therapeutic outcome.^10^, ^11^

Astaxanthin (ASX) and Sclareolide can both resensitize GEM‐resistant pancreatic cancer cells to GEM by downregulating RR expression as well as upregulating the uptake membrane proteins hENTs through a different signaling pathway. Therefore, they may be a novel agent in combination with GEM for the treatment of GEM‐resistant pancreatic cancer.^12^, ^13^

Liang et al reported that combination of LY2603618 (CHK1 inhibitor) with GEM synergistically overcomes chemoresistance of PDAC by downregulating RRM1/2 and promoting CDK‐dependent DNA damage.^14^

## 3 DRUG TRANSPORT

The effects of GEM are dose‐/concentration‐dependent and are mediated by transporters to cross extracellular or intracellular membranes. The two main transporter superfamilies are the solute carrier (SLC) superfamily and the ATP‐binding cassette (ABC) superfamily. Upregulation of drug uptake SLC transporters or downregulation of drug efflux ABC transporters are promising targets in GEM treatment of pancreatic cancer, but the later is more common strategy to overcome drug resistance.

Dauer et al^15^ showed that IT‐139 (inhibitor of glucose regulatory protein78) can not only decrease ABC transporters but also increase reactive oxygen species (ROS) by inhibiting antioxidant responses in PDAC.

Hsu et al reported that arginine methyltransferase 3 (PRMT3) binds to *ABCG2* mRNA to upregulate ABCG2, which increases PDAC resistance to GEM. Thus, PRMT3 inhibitors and GEM could be used as a cotreatment for pancreatic cancer in the future.^16^

Previous studies have reported that histone deacetylases (HDACs) are overexpressed in PC, and inhibiting HDACs could induce apoptosis and curb metastasis.^17^ A promising novel HDAC inhibitor, CG200745, can inhibit pancreatic cancer cell proliferation by increasing tumor suppressor genes and decreasing *ABCC3/ABCC4* genes.^18^ Paradoxically, HDAC inhibitors can enhance ABC transporters, thereby attenuating certain drugs.^19^ Accordingly, HDAC inhibitors could be combined with inhibitors of ABC transporters in PDAC.

ABC transporter inhibitor may be a novel target to overcome chemoresistance by increasing the concentration of the conventional anticancer drugs in cancer cells without producing additional toxicity.

## 4 PDAC SIGNALING PATHWAYS

Chemoresistance of PDAC are largely attributable to numerous signaling pathways, such as the JAK/STAT, Hedgehog, PI3K, RAS, nuclear factor (NF)‐kB, c‐Met, WNT‐*β*‐catenin, Notch, TGF‐*β*, SMAD, epidermal growth factor receptor (EGFR), mitogen‐activated protein kinases (MAPK), and SDF‐1/CXCR4 pathways (Table 1).

**Table 1:** Novel drugs target signaling pathways and RNAs

Novel therapies	Characteristic	Mechanism	Function
LY294002 and BI2536	PI3K/Akt and Polo‐like kinase 1 inhibitors	Signaling pathway	Enhance Chemosensitivity to Gemcitabine in PC
CUDC‐101	HDAC inhibitor	Signaling pathway	Reduce GEM‐resistance of PC
IONP‐LPrA2/DAPT	Leptin/notch inhibitor	Signaling pathway	Reduce chemoresistance of PC
Tetrakis and Octaacetyl	KRAS inhibitor	Signaling pathway	Reduce Epithelial to messenchymal transition
Nafamostat mesilate	NF‐*κ*B inhibitor	Signaling pathway	Enhance the antitumor effect of GEM/nPTX chemotherapy
Curcumin	NF‐*κ*B inhibitor	Signaling pathway	Enhance the anticancer activity of GEM in PC cells
SP600125	JNK inhibitor	Signaling pathway	Reduce chemoresistance on PC stem cells
AZD1480	JAK/STAT3 inhibitor	Signaling pathway	Enhance GEM delivery in PC
Caffeine	Akt phosphorylation inhibitor	Signaling pathway	Enhance chemo‐sensitivity of PC
Circ‐IARS	sponges miR‐122 (reduces its level)	Noncoding RNA	Enhance endothelial permeability and Inhibit chemo‐resistance

RAS, a frequently mutated G‐quadruplex forming proto‐oncogene, is a typical well‐known oncogene, and downregulation of *KRAS* has been proven to be associated with regulating the chemoresistance of pancreatic cancer. Notably, there are four downstream pathways of KRAS: PI3K/AKT, MAPK, RAL/GEF, and RIN1/ABL pathways. Growth‐factor receptors, such as EGFR, which stimulates the RAS/MAPK and PI3K/AKT signaling pathways, and VEGFR, can be overexpressed in PDACs, and signaling via these receptors activate RAS proteins by increasing receptor tyrosine kinase (RTK). RTK is upstream of the RAS/MAPK pathway (RAS/RAF/MEK/ERK/AP‐1).^20^

There are numerous, novel activating or inhibitory mechanisms that regulate the K‐RAS signaling cascade, decreasing chemoresistance as a result. Pattanayak et al^21^ demonstrated that porphyrins (Tetrakis and Octaacetyl) have a high affinity toward the G‐quadruplex of KRAS, exhibiting a significant ability to inhibit metastasis by blocking EMT and halting PDAC progression as a result. Cytokine tissue inhibitor of matrix metalloproteases (TIMP1) can bind to its receptor CD63 and then activate PI3K/AKT signaling. Inhibition of TIMP1 could improve the chemosensitivity of PDAC to GEM.^22^ Furthermore, Mao et al^23^ demonstrated that combined inhibition of the PI3K/AKT pathway (eg, LY294002) and inhibitors of Polo‐like kinase 1 (PLK1) (eg, BI2536) can enhance the GEM‐chemosensitivity of PDAC by inducing apoptosis in a nude mice model.

*AKT* is downstream of the Bitter taste receptor T2R10.^24^ Stern et al^25^ found that caffeine inhibited AKT phosphorylation and then reduced ABCG2 expression by triggering T2R10, which renders PDAC chemosensitive. Selumetinib and trametinib are superior MEK inhibitors that can reduce initial pancreatic volume.^26^ Inhibition of one mechanism may activate other compensatory protumor mechanisms that induce the chemoresistance of GEM. Therefore, inhibition of two mutually compensated chemoresistance mechanisms can dramatically improve chemosensitivity to GEM.

Mitogen‐activated protein kinases (MAPKs, also called extracellular signal‐regulated protein kinases (ERKs)) are downstream of the RTK/RAS/MAPK pathway.^27^

Ji et al demonstrated that CUDC‐101, a multitargeted inhibitor of HDAC, EGFR, and HER2, applied with GEM can not only induce apoptosis via PI3K/mTOR signaling, but also reduce GEM resistance of PC by inhibiting cell proliferation and EMT in PC via the ERK/Snail signaling pathway.^28^

The nuclear transcription factor kappa B (NF‐KB) pathway, which is a family of pleiotropic transcription factors associated with the regulation of immune and inflammatory responses, has a vital role in the development of PDAC.^29^ Inhibition of the NF‐KB signaling pathway can downregulate antiapoptosis downstream genes, representing a novel target for antiresistance in pancreatic cancer.^30^ Previous studies have proven that cyclooxygenase inhibitors such as sulindac and celecoxib can inactivate NF‐*κ*B, which as a result, reduces chemoresistance of PC.^31^ Recently, Pastorelli et al^32^ reinforced previous clinical evidence that curcumin (an inhibitor of NF‐*κ*B) can potentiate the anticancer activity of GEM in PC cells with low toxicity. They suggested that curcumin can be used as the first line therapy of advanced PC. Horiuchi et al found that a potent NF‐*κ*B inhibitor, nafamostat mesilate enhances the antitumor effect of GEM/nPTX chemotherapy for PC.^33^ Moreover, inhibition of tripartite motif containing 31 (TRIM31) could reduce NF‐*κ*B signaling and subsequently inactivate various antiapoptosis genes, which can enhance GEM efficiency in PDAC.^30^

Notch signaling has much more significance in PDAC by activating KRAS to promote EMT and tumor growth. Cui et al^34^ found that SNHG1 long noncoding RNA might reinforce the chemoresistance of PDAC by decreasing its apoptosis in cancer through the activation of the NOTCH signaling pathway. Inhibitors of SNHG1 could be an effective approach to chemotherapy. Leptin is vital for the activation of NOTCH signaling and induces the chemoresistance of PDAC. Recent clinical data showed that inhibition of leptin (eg, iron oxide nanoparticle‐leptin peptide receptor antagonist 2, IONP‐LPrA2)/notch (eg, gamma‐secretase inhibitor, DAPT) signaling can be used as a novel method to overcome the chemoresistance of PDAC.^35^

In conclusion, we found that different signaling pathways can interact with each other or share common pathways. This may be because they share a common effector. For example, MET and NOTCH can both activate the NF‐KB signaling pathway, and PI3K/AKT can be activated by RAS and MET. Therefore, we concluded that blocking downstream signaling pathways or downstream common pathways may be more effective than directly blocking upstream pathways. At present, there is insufficient experimental data to support this hypothesis, but it provides new ideas for future clinical trials.

## 5 APOPTOSIS AND CHEMORESISTANCE

Many studies of PC have demonstrated that chemoresistance can be explained by disturbances in apoptotic signaling pathways. There are two main apoptosis pathways. One is the extrinsic pathway, which is triggered by the Fas death receptor, the other is the intrinsic pathway, which leads to the release of cytochrome‐c from the mitochondria.

### 5.1 Novel therapy in the intrinsic pathway

One of the most important regulators of the apoptosis pathway is the BCL‐2 family, which includes proapoptotic members (BAX, BAK, BAD, and BID) and antiapoptotic members (BCL‐2, BCL‐XL, and BCL‐W).^36^

BCL‐XL is much more effective at preventing apoptosis than the other BCL‐2 family members. BCL‐XL is overexpressed in 90% of PDAC cases, and its expression level increases with the progression from PanIN‐1 to PDAC.^37^ On the contrary, overexpression of BAX can improve sensitivity to GEM.^38^ Therefore, the transcriptional regulator Yin Yang‐1 (YY1) becomes a potential druggable target for the development of pancreatic cancer treatments by directly activating *BAX* gene transcription.^39^

Lu et al^40^ found that verticillin A, a selective histone methyltransferase (HMTase) inhibitor, significantly increased the GEM sensitivity of human PDAC by increasing apoptosis (downregulating BCL‐x, FLIP, and MCL‐1, while enhancing BAK, BAX, and BIM). Moreover, melatonin and its metabolite N1‐acetyl‐N2‐formyl‐5‐methoxykynuramine enhanced the cytotoxic and tumor cancer cell proliferation inhibition effects of GEM in pancreatic cancer with upregulation of the BAX/BCL‐2 ratio and increased expression of active caspase.^41^

### 5.2 The extrinsic pathway

TRAIL, also called Apo‐2 ligand (Apo‐2L), belongs to the tumor necrosis factor family that preferentially triggers apoptosis in various tumor cells.^42^ It has drawn major attention for its potential utilization in cancer therapy as an effective anticancer tool that causes almost no cytotoxicity to normal cells. Combinations of TRAIL and certain DNA‐damaging drugs may have synergistic antitumor therapeutic effectiveness.

Spano et al used gene‐modified adipose mesenchymal stromal/stem cells (AD‐MSC) to stably secrete soluble TRAIL and induce rapid apoptosis, to demonstrate effective gene therapy for pancreatic cancer.^43^

Rossignoli et al further found that PTX can restore PC sensitivity to MSC‐delivered TRAIL and that the two compounds show improved cytotoxicity in pancreatic tumor cells, thus acting as a tool to improve treatment efficacy.^44^

In conclusion, the activation of proapoptotic genes and the blockade of antiapoptotic genes might enhance the chemosensitivity of pancreatic cancer.

## 6 AUTOPHAGY AND CHEMORESISTANCE

Autophagy is characterized as an intracellular self‐digestion process that is involved in the recognition and degradation of damaged proteins and organelles. It plays an important role in maintaining cellular homeostasis in response to stress. Autophagy has either prosurvival or prodeath functions in pancreatic cancer. For example, Onconase, a highly cytotoxic member of the pancreatic‐type ribonuclease (RNase) super‐family, strongly inhibits PDAC cell proliferation by triggering Beclin1‐mediated autophagic cell death and sensitizing cancer cells to GEM.^45^

Experimental evidence suggested that promotion of autophagy may sensitize cells to a cytotoxic effect, which leads to a better outcome for patients with pancreatic cancer.^46^ However, many studies also demonstrated autophagy provides resistance to treatment. One study reported that autophagy associates with the activity of pancreatic cancer stem cells and blockade of autophagy reduces pancreatic cancer stem cell activity and enhances the effect of GEM.^47^

Autophagy inhibitors include genetic inhibition (RNAi) and pharmacologic inhibition (chloroquine). Researchers reported that *miR‐29a* inhibits autophagy by blocking autophagy flux and downregulating autophagy proteins (TFEB and ATG9A), and contributes to reducing the invasive potential of pancreatic cells and sensitizing chemoresistant cancer cells to GEM.^48^

Recently, several experiments showed that many novel drugs focusing on autophagy regulate chemoresistance. 3‐MA has been reported to inhibit the activity of PI3‐Kinase and block the formation of preautophagosomes and autophagosomes.^49^ Linc‐RNA ROR (linc‐ROR) has been identified as an oncogenic lncRNA in pancreatic cancer, which confers GEM resistance to pancreatic cancer cells partly by inducing autophagy. And Linc‐ROR siRNA showed similar effect as 3‐MA in enhancing GEM sensitivity.^50^

Chloroquine (CQ) are commonly used in the treatment of malaria, but also are antiautophagy drugs in cancer treatment that raise intralysosomal pH and interfere with autophagosome degradation in the lysosomes.^51^ CQ can also inactivate pancreatic stellate cells (PSCs) by keeping them in a quiescent state and altering the tumor stroma, which reduces pancreatic tumor invasiveness.^52^ However, recent studies showed that CQ is not effective enough when applied alone. As above mentioned, gambogic acid sensitizes GEM efficacy but induces the autophagic process and promotes the survival of pancreatic cancer cells. Recent studies proved that CQ combined with gambogic acid can suppress pancreatic cancer and inhibit gambogic acid‐induced autophagic cancer survival.^53^

Thakur et al^54^ attempted to determine the combination effects of inhibitors of endoplasmic reticulum stress and autophagy applied with GEM in animal models. They concluded that triplet combination of chemotherapy (GEM + paclitaxel) + sunitinib + chloroquine showed the highest survival rate, indicating that the combination of autophagy inhibitors with GEM‐associated chemotherapy would be a novel therapy in PDAC.

Ubiquitin specific peptidase 9X (USP9X) is correlated with GEM resistance. Moreover, an inhibitor of USP9X, WP1130, can inhibit GEM‐induced autophagy to sensitize pancreatic cancer cells to GEM.^55^

In conclusion, autophagy has dual roles in pancreatic cancer progression, acting as a tumor suppressor or tumor protector. Targeting autophagy by promoting cell death in tumors and avoiding its survival mechanisms are novel therapies to overcome chemoresistance.

## 7 TUMOR MICROENVIRONMENT

The tumor microenvironment (TME) consists of both cellular and noncellular components, containing nonquiescent PSCs, immune cells, endothelial cells, fibroblasts, and extracellular matrix (ECM).^56^ These components provide pathological barriers hindering the delivery of drugs to cause chemoresistance.

However, the complex mechanisms of each component in promoting chemoresistance have been implied. Although TME hinders the delivery of drugs, some components can also contribute to tumor inhibition. Blindly depleting TME to increase the concentration of GEM in the tumor tissue may produce unpredictable consequences. Therefore, the current research direction is to specifically remove those components that can promote tumors. In other words, we can decrease pathological barriers using more selective strategies to preserve the tumor‐inhibiting components. An alternative approach is to inhibit the tumor‐promoting effect caused by TME depletion via drug combinations (Table 2).

**Table 2:** Novel drugs target microenvironment and stem cells

Novel therapies	Characteristic	Mechanism	Function
PX‐478	HIF‐1α inhibitor	CSCs	Enhance gemcitabine treatment in PC
Bethanechol	Muscarinic agonist	CSCs	Reduce pancreatic tumorigenesis and cancer stemness
Metformin	Oxidative metabolism modulator	CSCs	Cause fatal energy crisis in CSCs
4‐MU	UGT competitor	TME	Reduce hyaluronan synthesis and cell migration in PC
Hyaluromycin	HYAL inhibitor	TME	Reduce hyaluronan synthesis and cell migration in PC
Minnelide	Prodrug of triptolide	TME	Reduce hyaluronan synthesis and cell migration in PC
GW4869	Exosome release inhibitor	TME	Reduced survival of PC
Pasireotide	Somatostatin analogue	TME	Reduce PC chemoresistance
LB‐MSNP	GEM/PTX codeliverer/CDA inhibitor	Metabolism	Enhance PC chemosensitivity
Gambogic acid	RRM2 inhibitor	Metabolism	Enhance the efficacy of gemcitabine in PC
miR‐608 or miR‐101‐3p	RRM1 inhibitor	Metabolism	Enhance the efficacy of gemcitabine in PC
Astaxanthin and Sclareolide	RR and hENTs inhibitors	Metabolism	Enhance the efficacy of gemcitabine in PC
LY2603618	RRM1/2 inhibitor	Metabolism	Enhance the efficacy of gemcitabine in PC

### 7.1 Noncellular components

Extracellular matrix, mainly consists of collagen, noncollagen glycoprotein, heparan sulfate, and glycosaminoglycans. In PDAC, ECM is desmoplastic with abnormal accumulation of collagen fibers, leading to decreased delivery of drugs. However, previous studies depleting collagen fibers have had disappointing outcomes.

In recent years, hyaluronic acid (HA), another important component of the TME, has gradually attracted the attention of scientists. As HA within tumor tissue can bind water molecules, researchers speculated HA may exert pressure on surrounding structures and cause pathological barriers. Provenzano et al^57^ combined GEM with PEGPH20, a drug‐depleting HA, and observed significantly prolonged median survival (median overall survival of GEM‐ (55.5 days) and GEM + PEGPH20 (91.5 days)‐treated mice were significantly different (*P* = 0.004); treatment with PEGPH20 alone showed a trend toward increased survival (median = 63 days) that did not reach statistical significance (*P* = 0.1)). Importantly, PEGPH20 combined with GEM had significant therapeutic benefit on PDAC.

Another promising strategy is to inhibit HA synthesis. 4‐Methylumbelliferone (4‐MU), a competitive substrate for UDP‐glucuronosyltransferase (UGT), can inhibit HA synthesis by depleting cellular UDP‐GlcUA, and also downregulate HAS2 and/or HAS3.^58^ Hyaluronidase (HYAL) has also been demonstrated to be related to HA synthesis. Therefore, hyaluromycin, a HYAL inhibitor, was developed to decrease the concentration of LMW‐HA, and good results have been observed.^59^ Minnelide, which can also decrease the synthesis of HA, has been proved to result in better drug delivery in multiple animal models of pancreatic cancer. Notably, Minnelide is currently undergoing phase I clinical trials.^60^

Moreover, Chauhan et al^61^ demonstrated that HA‐induced vascular dysfunction is largely correlated with collagen. Therefore, they used losartan, a dual inhibitor of stromal collagen I and hyaluronan production by downstream inactivation TGF‐*β*1 to reduce solid stress, resulting in reduced tumor vessels. Increased drug and oxygen delivery can be observed with significantly improved chemotherapy outcomes.

### 7.2 Cellular components

CAFs (Cancer‐associated fibroblasts) produce type I collagen. Recently, Biffi et al^62^ demonstrated two CAF subtypes: myofibroblastic phenotypes (myCAFs) and inflammatory phenotypes (iCAFs). iCAFs have been shown to have potential tumor‐promoting components, whereas myCAFs can directly modulate EMT (Epithelial–mesenchymal transition) of PDAC cells, further restraining tumor progression.^63^

Previously, Özdemir et al^64^ employed a genetic strategy to deplete *α*SMA + myofibroblasts, and a significantly decreased tumor collagen content was observed. However, in this model, GEM therapy did not result in improved overall survival, because increased tumor invasion associated with myofibroblast depletion was reported.

Considering the heterogeneity of CAFs, more selective new therapeutic strategies targeting tumor‐promoting mechanisms may open the door to overcome CAF‐related chemoresistance.^65^ Richards et al^66^ reported that GEM‐exposed CAFs release exosomes to increase the chemoresistance‐inducing factor, Snail. They adopted GW4869 to inhibit exosome release, leading to significantly reduced survival of cocultured tumor cells. Duluc et al^67^ reported CAFs promote chemoresistance by secreting proteins such as IL‐6. They combined SOM230 analogue (Pasireotide) with GEM treatment to reduce IL‐6 production by inhibiting the protein synthesis mTOR/4E‐BP1 regulatory pathway, and with resulting reduced tumor growth and chemoresistance. As IL‐6 can lead to chemoresistance by activating JAK‐mediated STAT3 signaling, Nagathihalli et al^65^ combined GEM with AZD1480, a JAK/STAT3 inhibitor. This therapy enhanced drug delivery to the tumor without depletion of stromal collagen or hyaluronan, further improving therapeutic efficiency.

## 8 CANCER STEM CELLS

Recently, cancer stem cells (CSCs) have become a potential therapeutic target in PDAC. CSCs, which mainly remain in the G0 phase of the cell cycle, are naturally GEM‐resistant. Meanwhile, high expression of several important multidrug resistance transporter efflux chemotherapy drugs, such as ABCG2, has been reported on the surface of CSCs.^68^ A novel ABCG2 nonsubstrate antitumor molecule (FL118) reverses resistance to GEM in pancreatic cancer via complex mechanisms, one of which is reducing stem‐like pancreatic cancer cell populations.^69^

Moreover, the activation of CSC‐associated signaling pathways including MYC, JNK, AKT1, WNT/*β*‐catenin, NOTCH, and Shh, are also related to chemoresistance.^70^ Therefore, inhibiting these pathways is a promising strategy to increase the efficacy of chemotherapeutics. Suzuki et al^71^ reported that after treatment with JNK inhibitor SP600125, the effects of GEM on pancreatic CSCs were significantly enhanced with an increased proportion of dead cells.

Recently, researchers have relied on some characteristics of CSCs to design more‐targeted treatment drugs.

A recent survey demonstrated that EMT and the CSC phenotype are closely correlated because CSC generation is attributed to the activation of EMT. Indeed, the treatment of PDAC cells that have entered the CSC state with most conventional chemotherapeutics is rendered inefficient by activating EMT programme, promoting CSC‐dependent disease relapse as a result.^72^

### 8.1 EMT largely associated with CSCs

EMT can be defined as the phenotypic transition from an epithelial to a mesenchymal state. Previous studies demonstrated an inverse correlation between CSCs and EMT, which was largely associated with ZEB1 (zinc finger E‐box‐binding homeobox 1). Smigiel et al^73^ adopted shRNA‐mediated knockdown to silence *ZEB1*, resulting in an transition from mesenchymal/CSCs to epithelial/non‐CSCs. We can speculate that EMT has an important role in chemotherapeutic resistance, but the underlying mechanisms have yet to be elucidated. Zheng.et al^74^ reported that suppression of EMT enhanced the expression of nucleoside transporters, which contributed to enhanced sensitivity to GEM. As hypoxia inducible factor‐1 (HIF‐1) is highly associated with EMT, Zhao et al^75^ used PX‐478 to inhibit HIF‐1, which enhances the antitumor effect of GEM by inducing immunogenic cell death (ICD). All of this proves that targeting molecules and mechanisms associated with EMT may be a novel method to eliminate CSCs and improve chemosensitivity of GEM as a result.

### 8.2 Novel treatment specifically targeted at CSCs

A large amount of clinical data showed that some factors can greatly increase the number of CSCs in tumor tissues. Inhibiting these factors to specifically target CSCs has now become a promising therapeutic approach. The particularity of CSCs in metabolism compared with differentiated progenies in PDAC has recently received increased attention, and metabolism has become a new and feasible target. Whereas CSCs are reported to be highly dependent on oxidative metabolism,^76^ which means they are highly mitochondrial‐dependent. Thereby, Lonardo et al^77^ adopted metformin to target CSCs, observing a fatal energy crisis in CSCs through an increase in ROS production and a reduction in mitochondrial transmembrane potential. Resveratrol also rescues the stemness induced by GEM via suppression of sterol regulatory element binding proteins (SREBPs), which are members of a transcription factor family associated with the uptake and synthesis of cholesterol, fatty acids, and phospholipids.^78^

In conclusion, CSCs are closely associated with chemotherapeutic resistance and can be promising targets for overcoming chemoresistance. The current focus is on how to target CSCs more selectively, relying on specific characteristics of CSCs.

## 9 NONCODING RNA

Recently, noncoding RNAs (ncRNAs), including microRNAs (miRNAs) and long noncoding RNAs (lncRNAs), have been found to be associated with PDAC pathogenesis. miRNAs belong to small noncoding RNAs that have significant roles in cancer cell development, progression, and differentiation.^79^ Increasing evidence indicate that GEM resistance may be attributable to miRNAs in PDAC such as miR‐21, miR‐155, miR‐210, miR‐221, and miR‐222 and so forth.^80^ miRNAs degrade downstream mRNAs by binding to their 3'‐untranslated region (UTR). This mechanism influences the TME and contributes to EMT.^81^

An et al proved that human ovarian cancer‐specific transcript 2 (HOST2), a long noncoding RNA, could promote GEM resistance in human pancreatic cancer cells and that downregulated HOST2 could inhibit cancer cell proliferation and induce apoptosis of pancreatic cancer cells (Table 3).^82^ Moreover, You et al demonstrated that GEM decreases PVT1 (oncogenic long noncoding RNA) levels but increases its encoded miRNAs (*miR‐1207‐5p*/*miR‐1207‐3p*). Recently, Moschovis D et al conducted a case–control study to investigate the contribution of two lncRNAs polymorphisms of PVT1 and HOTAIR, respectively, in PC susceptibility and demonstrated that the PVT1 polymorphism was significantly overrepresented in PDAC patients compared with the controls.^83^ These findings implied that chemoresistance to GEM may partly be induced by lncRNA processing to miRNAs.^84^ However, Li et al reported that other lncRNAs such as lnc‐HOTTOP were associated with GEM sensitivity in PDAC.^85^

**Table 3:** Novel drugs target apoptosis and autophagy

Novel therapies	Characteristic	Mechanism	Function
IT‐139	Glucose regulatory protein78	Transpoter	Reduce GEM resistance of PC
CG200745	HDAC inhibitor	Transpoter	Reduce GEM resistance of PC
Yin Yang‐1	Bax gene activator	Apoptosis	Enhance PC cell apoptosis
Verticillin A	HMTase inhibitor	Apoptosis	Increase GEM sensitivity of PC
Melatonin	Upregulate Bax/Bcl‐2 ratio	Apoptosis	Increase GEM sensitivity of PC
FL118	Antiapoptotic proteins inhibitors	Apoptosis	Reverse resistance to GEM in PC
AD‐MSC	Soluble TRAIL producer	Apoptosis	Induce apoptosis in PC
miR‐29a	Autophagy inhibitor	Autophagy	Increase GEM sensitivity of PC
Linc‐ROR siRNA	Autophagy inhibitor	Autophagy	Increase GEM sensitivity of PC
CQ/HCQ	Autophagy inhibitor	Autophagy	Increase GEM sensitivity of PC
WP1130	USP9X inhibitor	Autophagy	Increase GEM sensitivity of PC

Notably, special lncRNAs such as circular RNA (circRNAs) can act as miRNA “sponges” to modulate targeted mRNA; circ‐RNAs are usually viewed as miRNA sponges as they include numerous conserved miRNA target sites.^86^ It has been demonstrated that circRNAs may be involved in the tumor development and GEM resistance of PDAC. For example, Xu et al found that circ_102747 could potentially bind *miR‐21*, a significant modulator in GEM resistance to PDAC through regulation of apoptosis by directly regulating BCL2 and PTEN expression.^87^ Moreover, Hao et al demonstrated that circ_0007534 regulates pancreatic cell proliferation, apoptosis, and invasion by sponging *miR‐625* and *miR‐892b*.^88^ In addition, Qu et al showed that circRHOT1 could bind *miR‐26b*, *miR‐125a*, *miR‐330*, and *miR‐382* to regulate multiple tumor‐associated pathways especially in PC.^89^

A promising mechanism of circular RNAs to decrease chemoresistance is physiologically increasing endothelial permeability. In human microvascular vein endothelial cells (HUVECs), activation of RAS homolog gene family member A (RhoA) signaling results in increased endothelial permeability, while *miR‐122* decreases RhoA signaling activity. Li et al^90^ revealed that circ‐IARS can enter HUVECs and sponges *miR‐122* to reduce its levels, leading to the activation of RhoA. This signaling pathway represents a novel, physiologically relevant mechanism to inhibit chemoresistance.

Above all, current studies have shown that noncoding RNAs participate in chemosensitivity and chemoresistance in pancreatic cancer. Notably, circRNAs were recently identified as a novel strategy for decreasing chemoresistance in patients with pancreatic cancer.

## 10 CONCLUSION

Hitherto, numerous potential chemoresistance mechanisms of PDAC have been elucidated. However, which mechanism has the dominant impact on PDAC chemoresistance and how to balance the effects of chemotherapy and toxicity remains unclear. In this article, we conclude that a reasonable combination of drugs that target different mechanisms generally improves chemosensitivity to GEM compared with monotherapy. In conclusion, our research aims should be focused on how to improve its efficiency and reduce toxicity. Combining those novel therapies with existing first‐line chemotherapy drugs may bring new hope for improving the survival of patients with pancreatic cancer.

## CONFLICT OF INTEREST

None declared.

## AUTHORS’ CONTRIBUTIONS

Study design: LWH; Literature search: LWH, LJY, and ZY; Study selection: LWH, LJY, and ZY; Study draft and revision: LWH, LJY, ZY, and YG; Article guarantor: Dr ZHANG Tai‐ping. The main contribution is completed by LWH.

## ETHICAL APPROVAL

Not required.

## DATA SHARING

No additional data available.
